# Yellow Mealworm Inclusion in Diets for Heavy-Size Broiler Chickens: Implications for Intestinal Microbiota and Mucin Dynamics

**DOI:** 10.3390/ani10101909

**Published:** 2020-10-18

**Authors:** Ilaria Biasato, Ilario Ferrocino, Elena Grego, Sihem Dabbou, Francesco Gai, Laura Gasco, Luca Cocolin, Maria Teresa Capucchio, Achille Schiavone

**Affiliations:** 1 Department of Agricultural, Forest and Food Sciences, University of Turin, Largo Paolo Braccini 2, 10095 Grugliasco (TO), Italy; ilaria.biasato@unito.it (I.B.); laura.gasco@unito.it (L.G.); lucasimone.cocolin@unito.it (L.C.); 2 Department of Veterinary Sciences, University of Turin, Largo Paolo Braccini 2, 10095 Grugliasco (TO), Italy; ilario.ferrocino@unito.it (I.F.); elena.grego@unito.it (E.G.); achille.schiavone@unito.it (A.S.); 3 Center Agriculture Food Environment (C3A), University of Trento, via E. Mach 1, 38010 San Michele all’Adige, Italy; sihem.dabbou@unitn.it; 4 Research and Innovation Centre, Fondazione Edmund Mach, 38010 San Michele all’Adige, Italy; 5 Institute of Science of Food Production, National Research Council, Largo Paolo Braccini 2, 10095 Grugliasco (TO), Italy; francesco.gai@ispa.cnr.it

**Keywords:** insect meal, microbiota, mucin, broiler chicken

## Abstract

**Simple Summary:**

Nowadays, the maximization of chicken productivity cannot be achieved without considering their gut health, which is a complex, multifactorial concept that takes into account several intestinal features (such as the microbiota and the mucin dynamics). The gut health of broilers may be influenced by both intrinsic (i.e., age, sex, breed) and extrinsic (i.e., diet, environment) factors, thus, in turn, influencing the growth performance of the birds. Dietary insect meal inclusion has already been reported to exert positive effects on cecal microbiota and small intestinal mucin composition in female and male light-size broiler chickens (35–40 days of age), in particular when used at low inclusion levels (i.e., 5%). However, since male heavy-size broilers (50–60 days of age) represents a relevant market class in Italy, we herein evaluated the effects of yellow mealworm (*Tenebrio molitor*, TM) utilization on their gut health. The findings herein obtained interestingly suggested that the administration of insect meal for a longer period could potentially lead to a negative modulation of the cecal microbiota of the birds, thus suggesting a preferable utilization of yellow mealworm in the light-size production cycles.

**Abstract:**

In the present trial, 160 heavy-size male broiler chickens were allocated to 4 dietary treatments (control feed [C] and 5, 10 and 15% TM meal inclusion, respectively, with 5 replicate pens/treatment and 8 birds/pen) to evaluate the influence of TM meal on intestinal microbiota and mucin composition. The broiler chickens fed TM-based diets showed higher beta diversity of their cecal microbiota in comparison with the C birds (*p* < 0.001). A significant decrease of the relative abundance of Firmicutes phylum and lower Firmicutes:Bacteroidetes ratios (False Discovery Rate [FDR] < 0.05) were also identified in TM15 broiler chickens when compared to the C group. Furthermore, the TM birds showed decreased relative abundance of *Clostridium, Coprococcus,* L-*Ruminococcus and Ruminococcus* genera (FDR < 0.05). In relation to the gut mucin composition, higher mucin staining intensity was detected in the intestinal crypts of TM5 birds in comparison with the other TM groups (*p* < 0.05). In conclusion, dietary TM meal inclusion negatively influenced the cecal microbiota of heavy-size broiler chickens in terms of partial alteration of the physiological microbial population and reduction of the potential beneficial bacteria (with slightly more pronounced effects when testing the 10–15% inclusion levels).

## 1. Introduction

It is well known that female and male broilers chickens are separately reared in the Italian production system, as the consumers seek three different market classes of birds: the so-called light-, medium- and heavy-size broilers. In particular, heavy birds are male broiler chickens that reach 3.4–3.6 kg of live weight during 54–58 day rearing cycles, in order to provide carcasses around 2.5- to 2.6-kg for the production of cut-up and further processed products [1]. Since almost 50% of the Italian male broiler chickens is usually slaughtered around 50–60 days of age, the scientific community has frequently focused its attention on this market class, as witnessed by several research studies [2,3,4]. The maintenance of a longer production cycle (54–58 days vs. 37–40 [light-size] and 45–52 [medium-size]) requires a particularly high feed efficiency, in order to optimise the overall productivity (which is a critical aspect in the poultry industry, since it is characterized by a shorter farming when compared to the other monogastrics).

Nowadays, it is impossible to maximise the chicken productivity without taking into account the concept of “gut health”, as a proper, effective gastrointestinal functionality has a crucial role in the determination of animal health, welfare and—most importantly—performance [5]. The key actor in the establishment of a good health status of the chicken gut appears to be the microbiota, which includes all the microorganisms identifiable in the gastrointestinal tract. Indeed, the gut microbiota strongly modulates the host physiological functions for the intestinal homeostasis maintenance (i.e., immune status, nutrient digestion, and intestinal mucosal barrier integrity), thus leading to both the competitive exclusion of potential pathogens and the saving of energy that is normally invested in keeping the immune system active against them [6]. Furthermore, the mucus layer—whose building blocks are highly glycosylated mucin proteins produced by goblet cells—is one of the main components of the intestinal mucosal barrier and represents the very first line of physical and functional defense against external molecules and pathogenic bacteria [7]. There is also an increasing evidence that dietary nutrients remarkably influence either the gut microbiota (in terms of modulation of synthesis of metabolites that directly affect the proliferation or the attachment of specific pathogens to the intestinal mucosa [5]) or the mucin dynamics (by altering mucin composition, mucus layer thickness, goblet cell staining, or mucin gene expression [8]). Therefore, the parallel characterization of the intestinal microbiota and mucin dynamics appears a fundamental approach when testing a novel, alternative feed ingredient in poultry diets.

The choice of an alternative feed ingredient should be influenced not only by its nutritional properties (thus, in turn, affecting the health status of the animals), but also by its capability of allowing a more sustainable use of natural resources and the safeguard of the environment. Insects easily meet all these conditions, because of all their remarkable nutritional profile (in terms of protein quantity and quality [9]) and environmental implications (in terms of low emission of greenhouse gas [10], the small land area needed to produce 1 kg of protein [11], and the ability to convert organic side streams into high-value protein products with advantageous feed conversion efficiency [12]). In the wake of such promising features, the scientific research on insect meal utilization in poultry, fish and pig nutrition has exponentially increased in the last decade, with a particular emphasis on nutrient digestibility, animal performance, gut health, and product quality [13,14]. Biasato et al. [15,16] have recently characterized the cecal microbiota and the small intestinal mucin composition in female and male light-size broiler chickens fed yellow mealworm (*Tenebrio molitor*, TM)—and black soldier fly (*Hermetia illucens*, HI)—based diets, observing overall positive findings with low inclusion levels (i.e., 5%). However, considering that host-related factors (including age) have been reported to widely affect the intestinal microbiota in poultry [17], the assessment of these key gut health parameters in heavy-size broiler chickens as well could provide novel insights into the practical adoption of insect-based products.

Based on these considerations, the present study aims to firstly evaluate the influence of dietary TM meal inclusion on intestinal microbiota and mucin composition of heavy-size broiler chickens.

## 2. Materials and Methods 

### 2.1. Birds and Experimental Design

The experimental design of the present study is reported in details by Biasato et al. [18]. In order to provide a brief summary, a total of 160 1-day-old male broiler chicks (Ross 708) were randomly allotted to four dietary treatments (five replicate pens/diet, eight birds/pen). The control diet (C) was based on corn meal, corn gluten meal, and soybean meal, while 5, 10 and 15% levels of full-fat TM larva meal (Gaobeidian Shannong Biology Co. Ltd., Gaobeidian, Hebei Province, China) were included to partially replace soybean meal, corn gluten meal and soybean oil to obtain the three experimental diets (TM5, TM10 and TM15, respectively). The chemical composition of the TM larva meal was as follows: 948 g/kg dry matter, 912 g/kg organic matter, 524 g/kg crude protein, 280 g/kg ether extract. Details of the diets are reported in Table S1. The growth performance of the broiler chickens were also evaluated throughout the experimental trial, as reported in details by Biasato et al. [18]. Briefly, the live weight (LW) and the average daily feed intake (DFI)—as well as the feed conversion ratio (FCR)—of the birds increased with increasing levels of dietary TM meal inclusion (LW: end of all the periods; DFI: starter and grower periods; FCR: finisher and overall periods). The experimental trial lasted 53 days. 

### 2.2. Intestinal Sampling and Processing

A total of ten birds per dietary treatment (two chickens/pen) were randomly selected and slaughtered in a commercial abattoir at the end of the experimental period. Cecal content, as well as small and large intestine segments, were sampled for DNA extraction (cecal content) and histochemical staining (gut segments), following the step-by-step procedures reported in details by Biasato et al. [19]. In particular, 5-cm-length, standardized gut segments of duodenum (the loop), jejunum (the tract before Meckel’s diverticulum), ileum (the tract before the ileocolic junction) and cecum (the apex) were collected [19].

### 2.3. DNA Extraction and Sequencing and Bioinformatics Analysis

A pool of the cecal content from two birds per pen (five pools per dietary treatment) was submitted to DNA extraction and sequencing. The DNA was extracted with a commercial kit (DNAzol^®^ Reagent, Thermo Fisher Scientific, Waltham, MA, USA) according to the manufacturer’s instructions. The cecal microbiota was then characterized by sequencing the amplified V3–V4 region of the 16S rRNA gene, according to the primers and PCR conditions previously reported [20]. Samples multiplexing, library purification and sequencing were performed as described in the “16S Metagenomic Sequencing Library Preparation” guide by Illumina Italy s.r.l. (Milan, Italy). As a final activity, all the libraries were sequenced by BMR genomics (Padova, Italy) on a MiSeq platform (Illumina Italy s.r.l., Milan, Italy), leading to 250bp, paired-end reads. QIIME 1.9.0 was used for data processing through the detailed pipeline previously reported [19].

### 2.4. Histochemical Staining

The formalin-fixed, paraffin-embedded intestinal sections of 10 chickens per diet (2 birds/pen) were submitted to three different histochemical staining, as previously reported by Biasato et al. [19]: periodic-acid Schiff (which identifies the neutral mucins in magenta), Alcian Blue pH 2.5 (that stains the acidic sialylated mucins in blue) and high iron diamine (which identifies the acidic sulfated mucins in purple-black).

### 2.5. Mucin Staining Intensity

The standardized, semiquantitative score recently adopted by Biasato et al. [19] allowed the assessment of the mucin staining intensity of the goblet cells on one slide per histochemical staining for each intestinal segment. In particular, a total of 10 crypts and 10 villi per each gut section were divided into three fragments (base, midsection and tip) and a 0–3 score was given to each fragment to determine the mucin staining intensity of the goblet cells [19].

### 2.6. Statistical Analysis

Alpha diversity indices were calculated using the diversity function of the vegan package [21]. Weighted UniFrac distance matrices were used to perform Adonis and ANOSIM statistical tests in the R environment (https://www.r-project.org). Diet-related differences were also assessed by pairwise *t*-test, Kruskal-Wallis tests or Wilcoxon rank sum test as appropriate. P-values were adjusted for multiple testing and a false discovery rate (FDR) <0.05 was considered statistically significant. A filtered OTU table was generated at 0.1% abundance in at least 2 samples through QIIME and used to build the Principal component analysis (PCA). The OTU table displayed the highest taxonomy resolution reached by the 16S data. Indeed, when the genus level was not reached by the taxonomy assignment, the bacterial family, order or phyla were actually showed.

The statistical analysis of the histochemical data was performed using IBM SPSS Statistics V26.0.0 software (Chicago, IL, USA). The histochemical scores were analyzed using the generalized linear model (GLM) recently adopted by Biasato et al. [19]. Results were expressed as least squares means and standard error of the mean (SEM). *p* values < 0.05 were considered statistically significant. A statistical trend was considered for *p* ≤ 0.10.

## 3. Results

### 3.1. Cecal Microbiota

The 16S rRNA sequencing allowed to obtain a total of 2,134,649 raw reads (2 × 250 bp), of which 669,570 reads passed the filters applied through QIIME with an average value of 33,479 reads/sample. All the samples were rarefied at 3600 reads after raw read quality filtering, in order to avoid biases due to the different sequencing depths. According to the rarefaction analysis and the Good’s coverage, all the samples were satisfactorily covered (average Good’s coverage of 86%, Table S2). 

**Figure 1:**
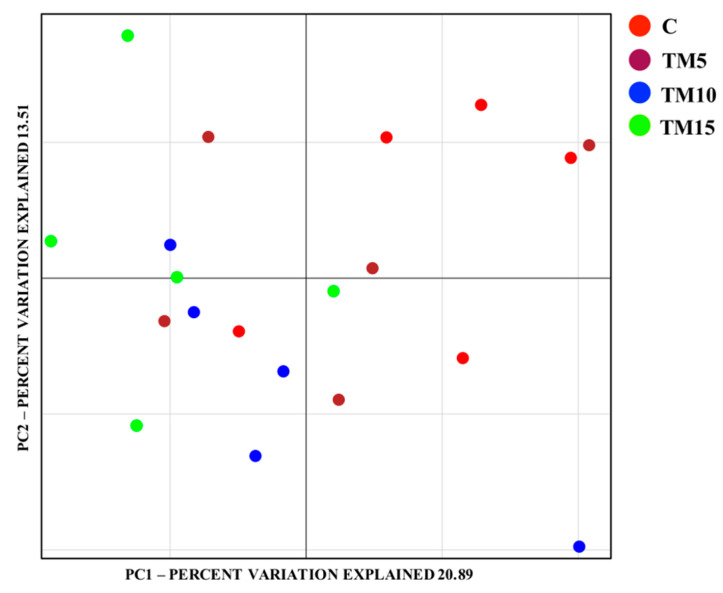
Bacterial community composition (weighted UniFrac beta diversity, principal component analysis plots) in cecal samples of male broiler chickens fed with control (C), 5% (TM5), 10% (TM10) and 15% (TM15) inclusion level of *Tenebrio molitor* meal diets.

No significant differences were identified between the α-diversity measures (Chao1, Phylogenetic Diversity [PD] Whole Tree and Shannon indexes, and observed species richness) of the C and the TM-fed birds (*p* > 0.05, Table S2). However, a clear separation of the microbial communities as a function of dietary TM meal inclusion (Adonis and ANOSIM statistical tests based on Weighted UniFrac distance matrix, *p* < 0.001) was displayed by the principal component analysis (PCA) (Figure 1). 

**Figure 2:**
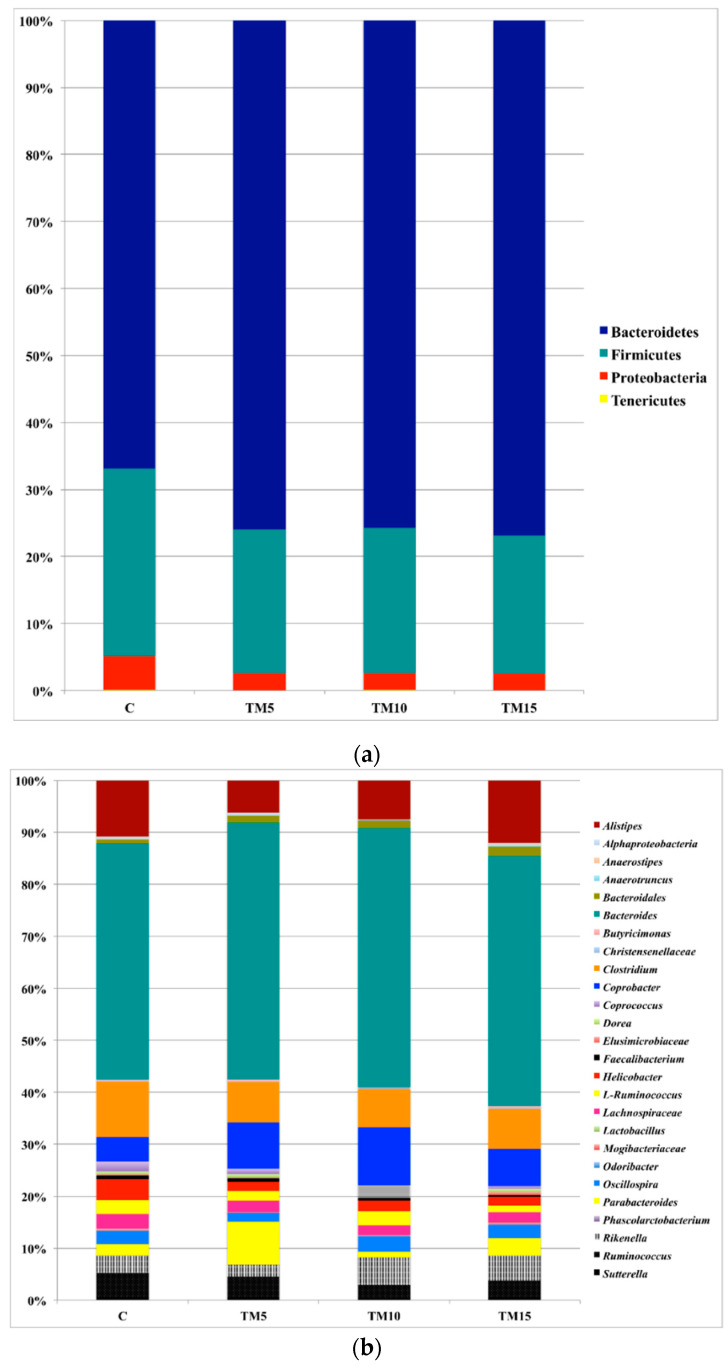
Relative abundance of the main bacterial phyla (**a**) and genera (**b**) in cecal samples of male broiler chickens fed with control (C), 5% (TM5), 10% (TM10) and 15% (TM15) inclusion level of *Tenebrio molitor* meal diets.

The cecal microbiota of either the C- or the TM-fed groups was dominated by the phylum Bacteroidetes, followed by Firmicutes and Proteobacteria (Figure 2a, Table S3). *Bacteroides*, *Alistipes*, *Coprobacter*, *Parabacteroides* and *Rikenella* were identified as main OTUs of the phylum Bacteroidetes in the birds fed both the C and the TM-based diets (Figure 2b, Table S3). Within the phylum Firmicutes, *Clostridium*, *Ruminococcus*, L-*Ruminococcus* (*Ruminococcus* genus belonging to the Lachnospiraceae family), *Oscillospira* and unclassified members (U. m.) of Lachnospiraceae family were the predominant genera in either the C- or the TM-fed groups (Figure 2b, Table S3). *Helicobacter* was observed as main OTU of the phylum Proteobacteria in the animals fed both the C and the TM-based diets (Figure 2b, Table S3). 

**Figure 3:**
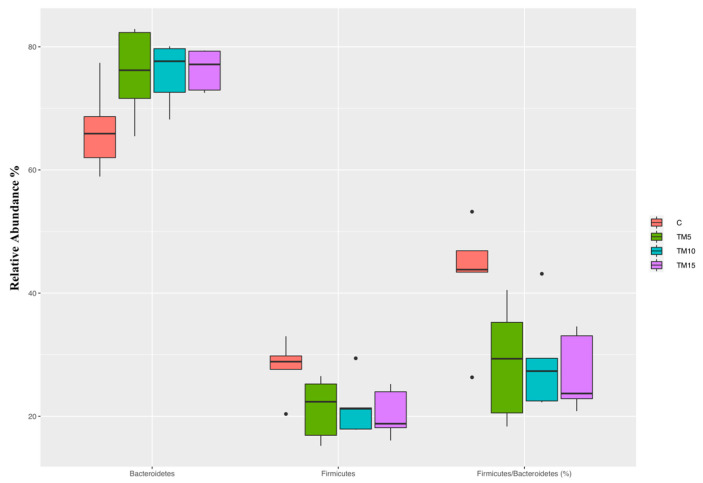
Phyla changes (FDR < 0.05) in cecal samples of heavy-size broiler chickens fed control (C), 5% (TM5), 10% (TM10) and 15% (TM15) inclusion level of *Tenebrio molitor* meal diets.

A significant decrease of the relative abundance of Firmicutes phylum—as well as lower Firmicutes:Bacteroidetes ratios—were identified in the TM15 broilers when compared to the C group (Figure 3, FDR < 0.05). The relative abundance of Bacteroidetes was, however, unaffected by dietary TM meal inclusion (Figure 3, FDR > 0.05).

**Figure 4:**
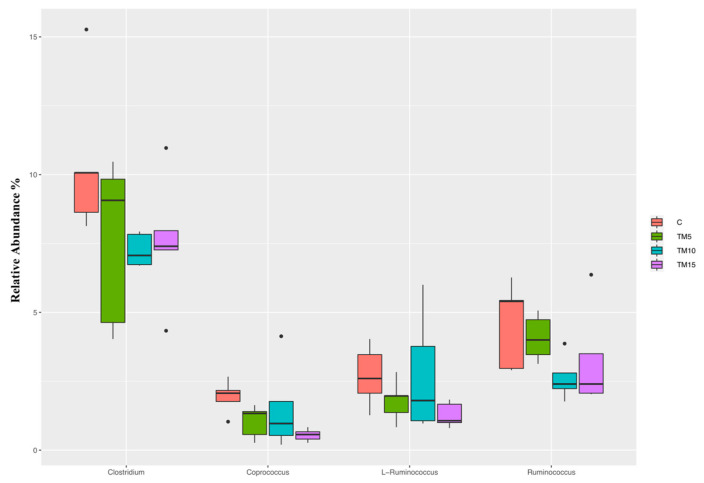
Genera changes (FDR < 0.05) in cecal samples of heavy-size broiler chickens fed control (C), 5% (TM5), 10% (TM10) and 15% (TM15) inclusion level of *Tenebrio molitor* meal diets.

As far as genus level is concerned (Figure 4), the relative abundance of *Clostridium*, *Coprococcus*, L-*Ruminococcus* and *Ruminococcus* was lower in animals fed the TM-based diets than the C (FDR < 0.05). The TM10 and TM15 birds displayed further significant decrease of the relative abundance of *Ruminococcus* compared to the C and the TM5 groups (FDR < 0.05).

### 3.2. Intestinal Mucin Composition

Dietary TM meal inclusion (*p* < 0.01), the mucin type (*p* < 0.001), the gut segment (*p* < 0.001) and the crypt fragment (*p* < 0.001) exerted a significant influence on the mucin staining intensity in the intestinal crypts of the male broilers (Table 1 and Table S4). In particular, TM5-fed birds showed higher mucin staining intensity in their crypts when compared to the TM10 and the TM15 (*p* < 0.01, Table 2). Furthermore, a predominance of the acidic sialylated mucins over the neutral and the acidic sulfated was revealed (*p* < 0.001, Table 2). The cecum and the jejunum also showed lower mucin staining intensity in their crypts than the other gut segments and the duodenum, respectively (*p* < 0.001, Table 2). Higher mucin staining intensity was finally identified in the crypt base when compared to the midsection and the tip (*p* < 0.001, Table 2). 

The mucin staining intensity in the intestinal villi was significantly influenced by the gut segment and the villus fragment (*p* < 0.001), with a statistical trend only being revealed for the mucin type (*p* < 0.10, Table 1 and Table S4). On the contrary, there was no significant effect of dietary TM meal inclusion (*p* > 0.05) on the histochemical findings (Table 1). In particular, the villi showed greater mucin staining intensity in the ileum than the other gut segments and in the jejunum than the duodenum, respectively (*p* < 0.001, Table 2). Furthermore, lower mucin staining intensity was observed in the villus tip when compared to the midsection and the base (*p* < 0.001, Table 2). The villi also showed a predominance of acidic sialylated mucins over the acidic sulfated (*p* < 0.001, Table 2).

## 4. Discussion

### 4.1. Cecal Microbiota

The broiler chickens fed either the C or the TM-based diets revealed Bacteroidetes, Firmicutes and Proteobacteria as main bacterial phyla in their cecal microbiota, thus agreeing with the previous reports in healthy chickens [22,23,24,25]. The predominance of the phylum Bacteroidetes over Firmicutes and Proteobacteria is also in agreement with the recent research assessing the effects of TM meal utilization on the gut microbiota of light-size female broilers [15]. On the contrary, Biasato et al. [16] recently observed that Firmicutes clearly outnumbered Proteobacteria and Bacteroidetes in light-size male birds fed HI-based diets. In regards to the genera profile, the cecal microbiota of the broiler chickens fed both the C and the TM-based diets showed *Bacteroides*, *Clostridium*, *Alistipes* and *Coprobacter* as predominant OTUs. These findings perfectly fit within the currently available literature about the physiological cecal microbial communities in chickens [24,26,27,28,29], being also in agreement with what was reported by Biasato et al. [15] in TM-fed light-size female broilers. In line with phyla characterization, Biasato et al. [16] identified, however, a predominance of Clostridiales order, members of Ruminococcaceae, *Faecalibacterium* and *Oscillospira* in the cecal microbiota of light-size male broilers fed diets containing HI meal.

As already reported by previous studies characterizing the cecal microbiota of light-size broilers fed diets containing TM meal [15,30], no differences were found in regards to α-diversity measures between the C- and the TM-fed birds of the present study. On the contrary, birds fed 15% level of HI meal inclusion recently displayed a lower Shannon index when compared to the C, 5% and 10% diets [16]. Concerning β-diversity, a clear separation of the cecal microbiota as a consequence of dietary TM meal inclusion was, instead, detected. This result is in agreement with previous researches about TM [15,19,31] and HI [16] meal utilization in diets for laying hens [31], free-range chickens [19], and light-size broiler chickens [15,16]. This scenario clearly confirms the strong predisposition of insects to modulate the complexity of the chicken intestinal microbiota, thus overall attenuating the partial decrease in α-diversity observed with the highest level of HI meal inclusion [16]. 

The broiler chickens fed the 15% level of TM meal inclusion showed a decreased abundance of Firmicutes phylum and lower Firmicutes:Bacteroidetes ratios when compared to the C diet, as similarly reported by Biasato et al. [15] in the light-size female birds fed diets containing 10 and 15% levels of TM meal inclusion. Since Firmicutes phylum is correlated with good intestinal health [32] and high Firmicutes:Bacteroidetes ratios are indicative of microbial communities with great capacity of energy harvesting [33], lower insect levels seem to be preferable for a better modulation of the gut microbiota (as already suggested by Biasato et al. [15,16]). 

Independently of the inclusion levels, the broiler chickens fed the TM-based diets in the present study showed a decrease in the abundance of *Clostridium*, *Coprococcus*, L-*Ruminococcus* and *Ruminococcus* genera in their cecal community. The majority of these taxa (*Clostridium*, L-*Ruminococcus*, and *Ruminococcus*) are characteristics members of the physiological chicken microbiota [24,26,27], being also involved in the production of metabolites that are fundamental for the health status of the gut barrier. Indeed, *Clostridium* and *Coprococcus* genera encompass bacteria capable of producing butyric acid [34,35], whose anti-inflammatory properties exert a positive role on bird growth, intestinal villus morphology, and pathogen concentrations [36]. Furthermore, *Ruminococcus* has a key role in the production of other short chain fatty acids (SCFAs; such as acetic and propionic acid) by exploiting glucose metabolism and cellulose digestion [37]. In turn, the produced SCFAs are capable of promoting the intestinal health by providing energy for enterocytes [38] and suppressing the potential pathogens [39]. Therefore, the decrease of *Clostridium*, *Coprococcus*, L-*Ruminococcus* and *Ruminococcus* above discussed suggests that TM meal utilization may negatively modulate the cecal microbiota of heavy-size broiler chickens. Previous studies characterizing the cecal microbiota of TM-fed light-size broilers revealed, however, overall positive insect-related effects in terms of increased abundance of potentially beneficial bacteria (such as *Alistipes*, *Sutterella*, and *Clostridium* [15], and *Ruminococcus* and *Lactobacillus* [30]). This contrasting outcome may be related to the different bird’s age (35 [30] and 40 [15] days vs. 53), as a partial confirmation of the wide influence of host-related factors on the poultry gut microbiota [17]. Biasato et al. [16] also reported inclusion level-dependent differences in cecal microbiota modulation by dietary HI meal inclusion, identifying potentially beneficial bacteria (such as L-*Ruminococcus*, *Faecalibacterium*, *Blautia*, *Clostridium*, *Ruminococcus*, *Lactobacillus*, *Bacteroides* and *Roseburia*) in all the HI-fed broiler chickens, but mucolytic, pathogenic bacteria (i.e., *Helicobacter*) in birds fed the 15% rate only. In the present study, despite the depletion of *Ruminococcus* genus being particularly evident in the broilers fed the TM10 and TM15, the different inclusion levels do not seem, however, to exert a clear, significant effect on the cecal microbiota of the animals. This scenario could probably reflect the differences in both the bird’s age (35 vs. 53 days) and the insect meal included (HI vs. TM), as a further confirmation of the complexity and multifactoriality of the poultry intestinal microbiota.

As a final consideration, it is well known that a clear cause-effect relationship between diversity and composition of cecal microbiota and bird performance is particularly difficult to be established. Biasato et al. [18] previously reported that increasing levels of dietary TM meal inclusion improved the body weight and the feed intake of the birds, but also impaired their overall feed efficiency. Therefore, since the above-mentioned, potential negative gut microbiota findings were overall identified independently of the TM meal inclusion rates, it appears unlikely that the microbiota changes alone had a remarkable impact on the overall growth performance of the animals. However, the broilers fed 15% level of TM meal inclusion showed the worst gut morphology in terms of shorter villi, deeper crypts and reduced villus height to crypt depth ratios [18]. Therefore, it is reasonable to hypothesize a parallel effect of both the gut microbiota and the morphology alterations on the animal performance, as an interesting confirmation of the complexity of the “gut health” concept.

### 4.2. Intestinal Mucin Composition

The intestinal crypts of the broiler chickens fed the 5% level of TM meal inclusion showed higher mucin staining intensity than the 10 and 15%, as similarly reported by Biasato et al. in the intestinal villi from TM- [15] and HI- [16] fed light-size birds. Considering the absence of mucolytic bacteria potentially explaining the increase in mucins as defense strategy [40], this finding appears related to the TM meal capability of preserving the positive properties of the mucins (i.e., modulation of digestion and absorption of nutrients, and removal of pathogenic bacteria [41]) at low inclusion rates. Furthermore, independently of dietary TM meal inclusion, the intestinal crypts of the broiler chickens showed a predominance of the acidic sialylated mucins over the other subtypes, thus representing a positive outcome as sialic acid groups are known to possess protective properties [42]. Since both the neutral and the acidic sialylated mucins have previously been reported to clearly outnumber the acidic sulfated (indicative of immature goblet cells [43]) in the gut crypts of TM- [15] and HI- [16] fed light-size broilers, it appears logical to conclude that insect meal utilization allows the preservation of mature, properly-developed mucin secretory dynamics. As a final aspect to consider in relation to the intestinal crypts, broiler chickens fed either C or the TM-based diets also showed lower mucin staining intensity in their caecum in comparison with the other gut segments. This finding is in agreement with what previously observed in light-size broilers [8,15] and free-range chickens [19], thus being probably related to the peculiar anatomy and physiology of the caecum [19].

Independently of TM meal utilization, the ileum of the broiler chickens showed greater mucin staining intensity in its intestinal villi when compared to the other gut segments. The density of the goblet cells has already been reported to physiologically increase along the duodenal-ileal axis in chickens [15,16,19], as the bacteria seem to have a clear preference for colonizing the distal ileum [40]. Therefore, this scenario potentially represents the need for greater protection and subsequent higher mucin synthesis [40]. The base of the intestinal crypts and villi of the broiler chickens fed either the C or the TM-based diets also showed greater mucin staining intensity in comparison with the other fragments, thus reflecting the physiological processes of proliferation and maturation of the goblet cells occurring in crypt [8,15,16,19] and villus [15,19] compartments.

## 5. Conclusions

In conclusion, dietary TM meal inclusion negatively modulated the cecal microbiota of heavy-size broiler chickens, as a partial alteration of the physiological microbial population and a reduction of the potential beneficial bacteria (with slightly more pronounced effects when testing the 10–15% inclusion levels) were identified. However, diets containing yellow mealworm can still maintain the positive properties of the mucins in the gut. This scenario interestingly underlines that the administration of TM meal to the broiler chickens for a longer period (which is characteristic of the heavy-size market class) could potentially lead to microbial alterations, even if further investigations are mandatory to confirm this hypothesis. Nevertheless, the identification of a physiological cecal community and gut mucin dynamics in all the birds (independently of dietary TM meal inclusion) overall attenuates the observed negative outcomes.

## Figures and Tables

**Table 1:** Influence of the considered predictor factors (diet, mucin type, gut segment and crypt-villus fragment) on the gut mucin staining intensity in the heavy-size broiler chickens.

Factor	d.f. ^6^	Chi-Square	*p* ^7^
Crypts			
Diet ^1^	3	12.388	0.006
Mucin type ^2^	2	18.860	<0.001
Gut segment ^3^	3	75.407	<0.001
Fragment ^4^	2	96.076	<0.001
Villi			
Diet	3	4.045	0.257
Mucin type	2	4.937	0.085
Gut segment ^5^	2	748.764	<0.001
Fragment	2	43.135	<0.001

^1^ Four dietary treatments: C = control; TM5 = 5% inclusion level of *Tenebrio molitor*; TM10 = 10% inclusion level of *Tenebrio molitor*; TM15 = 15% inclusion level of *Tenebrio molitor*. ^2^ Three types: neutral, acidic sialylated and acidic sulfated mucins. ^3^ Four gut segments: duodenum, jejunum, ileum and caecum. ^4^ Three fragments: base, midsection and tip. ^5^ Three gut segments: duodenum, jejunum and ileum. ^6^ Degrees of freedom. ^7^ Statistical significance: *p* < 0.05.

**Table 2:** Mucin staining intensity in the intestinal crypts and villi of the heavy-size broiler chickens according to the specific predictor factors (diet, mucin type, gut segment and fragment).

Mucosal Element	Predictor	Predictor Factors	Mucin Staining Intensity ^1,2^
Crypts	Diet	C	0.98 ± 0.02 ^ab^
		TM5	1.04 ± 0.03 ^a^
		TM10	0.94 ± 0.02 ^b^
		TM15	0.93 ± 0.02 ^b^
	Mucin type	Neutral	0.93 ± 0.02 ^b^
		Acidic sialylated	1.05 ± 0.02 ^a^
		Acidic sulfated	0.96 ± 0.02 ^b^
	Gut segment	Duodenum	1.09 ± 0.03 ^a^
		Jejunum	0.99 ± 0.02 ^b^
		Ileum	1.03 ± 0.02 ^ab^
		Cecum	0.82 ± 0.02 ^c^
	Fragment	Base	1.15 ± 0.02 ^a^
		Midsection	0.89 ± 0.02 ^b^
		Tip	0.91 ± 0.09 ^b^
Villi	Diet	C	1.70 ± 0.04
		TM5	1.69 ± 0.04
		TM10	1.59 ± 0.04
		TM15	1.67 ± 0.04
	Mucin type	Neutral	1.68 ± 0.04 ^ab^
		Acidic sialylated	1.71 ± 0.04 ^a^
		Acidic sulfated	1.60 ± 0.04 ^b^
	Gut segment	Duodenum	1.01 ± 0.02 ^c^
		Jejunum	1.96 ± 0.04 ^b^
		Ileum	2.31 ± 0.05 ^a^
	Fragment	Base	1.80 ± 0.04 ^a^
		Midsection	1.71 ± 0.04 ^a^
		Tip	1.49 ± 0.03 ^b^

^1^ Data are represented as mean of counts ± SEM. ^2^ Means with different superscript letters (a, b, c) within the same column per predictor (i.e., diet, mucin type, gut segment or fragment) differ significantly (*p* < 0.01). C = control; TM5 = 5% inclusion level of *Tenebrio molitor*; TM10 = 10% inclusion level of *Tenebrio molitor*; TM15 = 15% inclusion level of *Tenebrio molitor*.

## Supplementary Materials

The following are available online at https://www.mdpi.com/2076-2615/10/10/1909/s1, Table S1: Feed ingredients and proximate composition of the experimental diets. Mineral-vitamin premix (Final B Prisma, IZA SRL, Forlì, Italy), given values are supplied per kg of diet: 2.500.000 IU of vitamin A; 1.000.000 IU of vitamin D3; 7.000 IU of vitamin E; 700 mg of vitamin K; 400 mg of vitamin B1; 800 mg of vitamin B2; 400 mg of vitamin B6; 4 mg of vitamin B12; 30 mg of biotin; 3.111 mg of Ca pantothenate acid; 100 mg of folic acid; 15.000 mg of vitamin C; 5.600 mg of vitamin B3; 10.500 mg of Zn, 10.920 mg of Fe; 9.960 mg of Mn; 3.850 mg of Cu; 137 mg of I; 70 mg of Se. AMEn = apparent metabolizable energy; CP = crude protein; EE = ether extract; NDF = neutral detergent fiber; ADF = acid detergent fiber; C = control; TM5 = 5% of *Tenebrio molitor*; TM10 = 10% of *Tenebrio molitor*; TM15 = 15% of *Tenebrio molitor*. Table S2: Good’s coverage and α-diversity measures of cecal microbiota of heavy-size broiler chickens fed control (C), 5% (TM5), 10% (TM10) and 15% (TM15) of *Tenebrio molitor* meal. Description column indicates the 5 replicate pens of control (C_1, C_2, C_3, C_4 and C_5), 5% (TM5_1, TM5_2, TM5_3, TM5_4 and TM5_5), 10% (TM10_1, TM10_2, TM10_3, TM10_4 and TM10_5) and 15% inclusion level of *Tenebrio molitor* meal (TM15_1, TM15_2, TM15_3, TM15_4 and TM15_5) dietary treatments. Table S3: Relative abundance of the main bacterial phyla and genera of cecal microbiota of heavy-size broiler chickens fed control (C), 5% (TM5), 10% (TM10) and 15% (TM15) of *Tenebrio molitor* meal. Table S4: Mucin staining intensity scores of heavy size broiler chickens fed control (C), 5% (TM5), 10% (TM10) and 15% (TM15) of *Tenebrio molitor* meal.
